# Doxofylline for Pediatric Asthma Steps 1–4. Pediatric Asthma: New Role for an Old Drug

**DOI:** 10.3389/fped.2022.772704

**Published:** 2022-06-22

**Authors:** Vincenzo Fierro, Anna Lucia Piscitelli, Edda Battaglia, Alessandro Fiocchi

**Affiliations:** ^1^Predictive and Preventive Medicine Research Unit, Multifactorial and Systemic Diseases Research Area, Bambino Gesù Children’s Hospital, Istituto di Ricovero e Cura a Carattere Scientifico (IRCCS), Rome, Italy; ^2^ABC Farmaceutici S.p.a., Ivrea, Italy

**Keywords:** asthalin drug, doxofylline, metilxantine, inhaled corticosteroid, pediatric

## Abstract

The panoply of anti-asthma drugs for children between 6 and 18 years is not limited to those reported in the guidelines. In this review, we will re-assess the role of doxofylline, a xanthine characterized by a much higher handling than that of theophylline, as add-on treatment in pediatric asthma grade 1–4. Ten studies evaluated doxofylline in the treatment of asthma of patients non-responsive to the first-line inhaled corticosteroids. Of these, two included children and one was exclusively pediatric. According to their results, doxofylline exerts a powerful bronchodilator and anti-inflammatory activity, which can be exploited when the inhaled oral corticosteroids are not sufficient to get the desired effect of reducing symptoms. Unlike theophylline, doxofylline does not require blood testing. It can be administered together with or as an alternative to a series of other drugs considered in additional therapy.

## Introduction

Affecting about one in 12 children, asthma is the most prevalent chronic pediatric disease (1).

The most frequent presentations are mild to moderate ones, which is “a non-severe asthma which can be controlled by steps 1–4 GINA treatment” (2). While healthcare costs (3), caregiver burden (4), impaired quality of life (5), and loss of school days (5, 6) are associated mainly with sever e asthma, children with mild-to-moderate forms may in turn incur non-negligible risks (4, 6). This form of asthma is subject to significant heterogeneity (7). Predictors of poor outcomes are still largely unknown (8), but children with asthma grade 1–4 are subject to reduced lung function (9), abnormal patterns of lung growth (10), risk of chronic obstructive pulmonary disease in adulthood (11), severe exacerbations (12), and even fatalities (13). This may be increased by the lower compliance to regular controller medication compared to severe asthma (14). We review here the therapy of asthma grade 1–4 in children in the light of the latest guidelines, with a reflection on the evolution of the guidelines over time. We will focus particularly on methylxanthines, a category of drugs that have been abandoned. We aim to verify the reasons for their abandonment, and the opportunities they may still offer today.

## Methods

We selected the following electronic databases:

• NCBI PubMed (1999 onward);
• EMBASE (1999 onward);
• UKCRN (the UK Clinical Research Network Portfolio Database);
• WHO ICTRP (the World Health Organization International Clinical Trials Registry Platform);
• The Cochrane Central Register of Controlled Trials;
• ISI Web of Science;
• Google Scholar.

Repeated searches were carried out using the template algorithm [asthma AND (…)] with the settings: (Humans; English; All Child 0–18; Clinical trial; Last 30 years) for the following comparators: methylxanthines; theophylline; doxofylline; aminophylline. The full-text versions of the studies were independently retrieved from the NIH PubMed database. The authors met following review and appraisal according to their clinical experience (VF, ALP, EB) and consultant-level management (VF, AF) of pediatric asthma in a pediatric tertiary-level institutional setting. The few studies retrieved were scored with trivial differences between clinicians and managers/editor and we thus opted for a narrative review.

### Oral Administration Increases Drug Compliance in Pediatric Asthma

The use of combined steroid—Long-Acting β-Agonist (LABA) inhalers has been largely adopted in the last guidelines as the result of studies indicating the superiority of steroid-LABA combination vs. the use of two regular medications in separate inhalers (15). This include cost-effectiveness (16), cost-utility (17), asthma control (18) and time to the first exacerbation (19). A non-negligible aspect of the use of combined inhalers is their positive effect on the patient’s compliance, largely resulting from an increased convenience with the combination inhaler (20).

Already 25 years ago, it was reported that the use of an oral route bid is clearly superior for compliance to the use of the inhalation routes, especially if divided into three or four administrations (Table 1) (21).

**TABLE 1:** Better compliance of the oral respect to the inhalation route (21).

Route of administration	Complete %	Partial %	Nihil %
Dry powder inhaler	52.3	35.8	5.5
MDI	66	7.6	7.6
Aerosol	48.9	18.9	10.8
Oral	76	9.2	3.7

**FIGURE 1:**
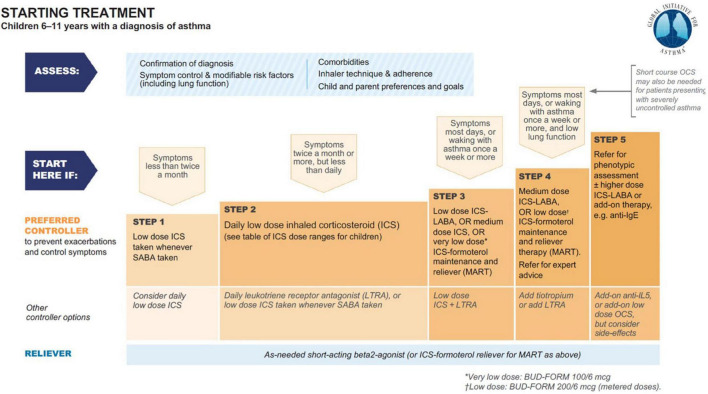
Asthma treatment from GINA 2021 guideline main report (24).

Starting from the 1980s, theophylline monotherapy was popular for the treatment of stable chronic asthma in children and adults, especially since the introduction of Slow-Release Theophylline made its use more manageable (22, 23). With the increase of use of topical corticosteroids, it became second line. From 2006, theophylline was progressively marginalized from the category of drugs for the treatment of asthma until it disappeared completely in the latest edition of the GINA guidelines (Figure 1) (24). Reasons for that essentially include lack of further studies, and its poor handling (25): due to the narrow therapeutic range of theophylline, and its potential side effects (see *infra*), the use of slow-release theophylline over the long term requires a periodical measure of theophylline serum levels in basal and peak conditions (26). Yet, the characteristics of the drug are surprisingly modern as, exactly as the combination budesonide-formoterol; it combines the bronchodilator action with the anti-inflammatory action.

### Methylxanthines and Theophylline in Pediatric Asthma

In 1859, Henry Hyde Salter MD described in the Edinburgh Medical Journal his personal experience as asthmatic patient. According to him, “… one of the most common and best-regarded remedies for asthma … is strong coffee” (27). From 1920 onward, the methylxanthines were identified as able to relax the smooth bronchial muscle *in vitro* (28). Theophylline, and its water-soluble derivative aminophylline, have since then been used in the treatment of asthma.

This molecule is the most potent bronchodilator methylxanthine, with anti-inflammatory and immunomodulatory activities (29). The molecular mechanism of bronchodilation is the inhibition of phosphodiesterase (PDE) 3 and PDE4. The anti-inflammatory effect has been attributed to histone deacetylase (HDAC) activation, resulting in switching off activated inflammatory genes. Theophylline is able to counteract corticosteroid resistance: this may be of particular value in severe asthma, where HDAC2 activity is markedly reduced. Because of these mechanisms, the drug improves the strength of respiratory muscles, improves mucociliary clearance, and stimulates the cerebral respiratory centers.

Side effects include headache, nausea, vomiting, abdominal discomfort, and restlessness. There may also be increased acid secretion, gastroesophageal reflux, and diuresis. At high concentrations, convulsions and cardiac arrhythmias have been reported (30). These effects are attributed to the effect of theophylline on adenosine receptors: theophylline is an inhibitor of A1- and A2-receptors, involved in the releases of histamine and other mediators from mast cells. Adenosine antagonism is likely to account for central nervous system stimulation, cardiac arrhythmias, gastric hypersecretion, gastroesophageal reflux, and diuresis (31).

**FIGURE 2:**
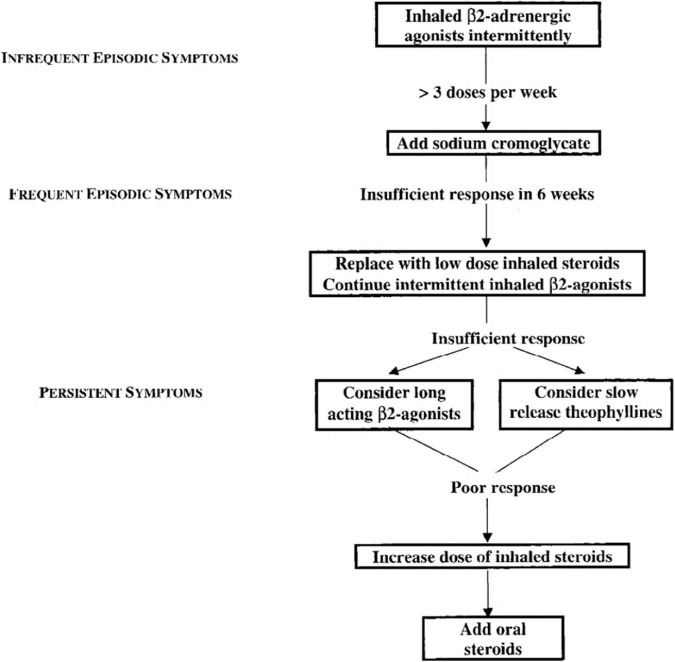
1998 International Pediatric Consensus statement on the management of childhood asthma – flow chart (33).

While the potential toxic effects of theophylline were already recognized, with the use of serum levels stabilization they became rare. There is evidence from many clinical studies that adding theophylline confers a benefit in patients with chronic asthma who are have already been treated with inhaled corticosteroids (32). On these premises, the 1998 International Pediatric Consensus statement on the management of childhood asthma prescribed the use of theophylline in all the situations of persistent asthma where low-dose inhaled steroids are not sufficient to control symptoms (Figure 2) (33).

### Doxofylline: Pharmacologic Characteristics

**FIGURE 3:**
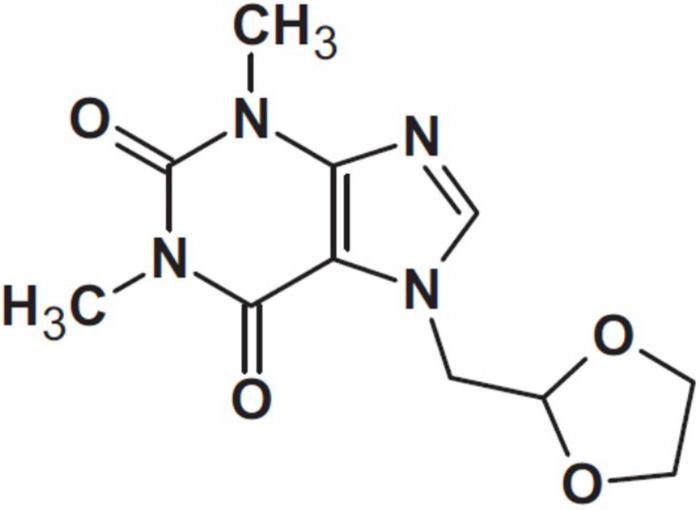
Doxofylline (34).

Doxofylline has been defined a “novofylline” (34). This xanthine, chemically designated as 7-(1, 3 dioxolar-2-ylmethyl)-theophylline, features a dioxolone group in position 7 (Figure 3).

Doxofylline is metabolized as a theophylline derivative by the liver, and eliminated by urinary excretion within 12 h after the administration. In contrast to theophylline, it does not interfere with the liver Cytochrome P450 enzymes CYP1A2, CYP2E1, and CYP3A4, implied in drug metabolism. Therefore, there is no interaction between doxofylline and any of the drugs that interfere with the cytochrome. This cancels the usage restrictions for Erythromycin, Roxithromycin, Enoxacin, Ciprofloxacin, Ofloxacin, Fluoroquinolone T-3262, Pipemidic acid, Cimetidine, Etintidine, Propranolol, Verapamil, Nifedipine, Furosemide, Allopurinol, Ticlopidine, Idrocilamide, Thiabendazole, Disulfiram, Isoproterenol, Terbutaline, Oral corticosteroids, Phenytoin, Phenobarbital, Benzodiazepines, and Sulfinpyrazone (35, 36).

Doxofylline peaks at 2 h and produces stable serum concentrations. The serum concentrations after administration of 100 mg as a single intravenous dose, or 400 mg orally (both twice daily for 5 days), are the following:

– i.v. doxofylline, peak serum concentration of 25.65 ± 3.98, half-life of 1.83 ± 0.37 h;
– doxofylline given orally, peak serum concentration of 15.21 ± 1.73 micrograms/ml with a half-life of 7.01 ± 0.80 h (37).

The maximum effects of is observed at 6 weeks for asthmatic patients. Due to lack of evidence of adverse event related to doxofylline blood levels, there is no need to monitor them during the therapy either using low-dose or high-dose. Thus, doxofylline does not require to be dosed in the blood at baseline and peak conditions; plasma monitoring is only required in patients with hepatic insufficiency and intolerance to methylxanthines (38).

### Therapeutic Effects

The drug retains all the pharmacologic activities of theophylline. It is able to exert the bronchodilator action through PDE3 and PDE4 inhibition, and the anti-inflammatory effects through HDAC activation. While in asthma Interleukin (IL)-10, a potent anti-inflammatory agent, is heavily reduced (39), doxofylline favors IL-10 release by PDE inhibition (40). Among the others studied mechanisms, it acts by inhibiting LPS-superoxide anion production in human monocytes during short treatments, while in longer treatments his action is mediated by modulation of Protein kinase C activity (41). As theophylline, doxofylline exhibits anti-inflammatory properties, but the specificity of the anti-inflammatory action of the two drugs is not identical. In addition, doxofylline links to β2-adrenoireceptors with hydrogen bond, eliciting relaxation of blood vessel, and bronchial smooth muscles (42).

### Side Effects

The safety and tolerance profiles of doxofylline have been explored in a large number of comparative studies. Compared to theophylline, doxofylline exhibits a reduced affinity for A1- and A2-adenosine receptors (43), reducing the side effects and contributing to the better safety profile. It does not antagonize calcium channels, and does not interfere with the influx of calcium into the cells (44). While all methylxanthines cause a significant increase in heart rate, doxofylline displays less cardiac activity compared to theophylline. In guinea pig right and left atrial preparations, and in anesthetized cat, doxofylline increased the atrial rate at 0.3 mM/L concentration, while theophylline induces positive chronotropic effect already at the dose of 0.03 mM/L (45).

All these characteristics give doxofylline a wider therapeutic window than theophylline (34).

### Clinical Trials: A Look From the Pediatric Point of View

A single trial, done in pediatric patients, demonstrated a clinical benefit of intravenous administration of doxofylline, 5 ml/kg, in 116 children with acute asthma attacks (46), but—as per theophylline—the use of doxofylline in asthma attacks is limited (25).

Conversely, we have so far nine published clinical trials of doxofylline as long-term controller in asthma. The first randomized, placebo-controlled study was a pediatric one (47). In a double/blind design, doxofylline was administered at the dose of 6 mg/kg every 12 h for 2 weeks to children aged 6–12 years. Statistically significant differences for FEV1, forced expiratory flow at mid-term of the forced vital capacity and PEFR were found in the treated vs. placebo group. The study group, evaluated at 7 and 14 days of treatment, showed the persistence of such improvement. No major side effects were reported. The main limitations of this study were the limited sample size and a short duration of follow-up.

Since then, the clinical studies have involved increasing numbers of patients (48–50).

Their conclusions shown the effectiveness of doxofylline in asthma, with an efficacy/safety profile better than that of theophylline (50). The number of children included is not substantial. In particular, no children were present in the LESDA study (51), in DOROTHEO1 and 2 (52), and in the Indian trial (53, 54). Two studies included pediatric patients, aged 15–18 and 12–18 years, respectively (55, 56). The conclusions drawn from these trials can thus be applied to the pediatric population with the caution dictated by extrapolation to pediatric age of studies on adult asthma (57).

More substantial is our information about the safety profile of doxofylline in pediatric age. A panel of 102 experts collected data on 806 pediatric patients aged 3–16 years affected by asthma. doxofylline was given as add-on therapy to beta2-agonists, mucolytic, corticosteroids, antibiotics, non-steroid anti-inflammatory drugs, at dose of 6 mg/kg two times daily using sachets of 200 mg each. This dose was increased up to 9 mg per Kg every 12 h if response was judged not satisfied. The authors reported a 11% side effect rate. The patients’ dropout related to side effects was 5%. The vast majority of side effects observed were related to the gastro- intestinal system (76%), although some were attributed to effects on the central nervous system (16%). The occurrence of palpitations was the only side effect attributed to the cardiovascular system (9%) and the tolerability of doxofylline was judged as satisfactory in most of the cases (76%) (58).

### Doxofylline vs. Theophylline

**FIGURE 4:**
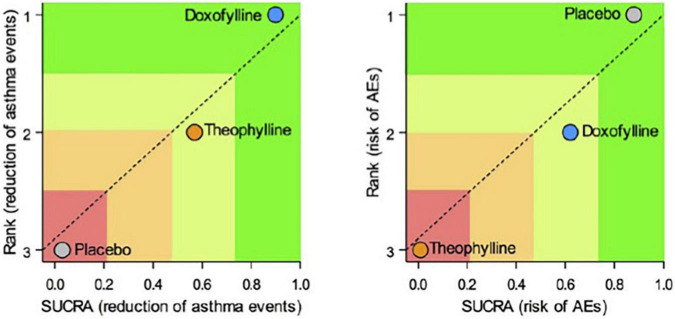
Safety and efficacy of doxofylline compaired to theophylline (59).

Doxofylline is not just another theophylline (34). In 2019, a quantitative synthesis compared the efficacy/safety profile of the two drugs in asthma. Elaborating on four available comparative studies, this meta-analysis showed that treatment with doxofylline was significantly more effective than theophylline in reducing the daily asthma events and preventing the risk adverse events (AEs) (Figure 4) (59). The percentage of the most frequently recorded AEs (headache, nausea, insomnia, dyspepsia, and vomiting) was greater in asthmatic patients treated with theophylline than in those that received doxofylline. In addition, doxofylline was found as effective as theophylline in improving FEV1. It was superior to theophylline concerning in reduction of rescue medication.

### Steroid Sparing Effect

The ability of doxofylline to reduce corticosteroids doses was indirectly assessed (60).

When it is used in association with glucocorticoids, their need is reduced. The difference was more significant in the over 65 aged group, both in men and women. The authors suggest that data is important considering that doxofylline is mainly prescribed to women who experience the highest osteoporosis damage after the corticosteroid therapy. One can infer that this is also the case for pediatric age, a time when the calcium/phosphorus equilibrium is of utmost importance (61).

### Doxofylline in the Pediatric Asthma Arsenal

Established treatment strategies for mild-to-moderate asthma (GINA steps 2–4) include allergen avoidance, inhaled corticosteroids (ICS), short- and long-acting β2-adrenoceptor agonists (SABA and LABA), CysLTR1 antagonists (LTRA; montelukast, zafirlukast, and pranlukast), long-acting muscarinic antagonists (LAMA), immunosuppressants (methoytrexate, azathioprine, cyclosporine), and chromones (sodium cromoglicate and nedocromil) (23). As the interventions vary by mechanism of action, effectiveness, side effects, feasibility, and cost, establishing the correct treatment for each case remains an art. In his partnership with the patient and her family, the praticizing physician takes in account a series of clinic and environmental considerations (62). This is the practical implementation of the guidelines, according to which the therapeutic interventions must be calibrated on the “values and preferences” of the clinician, the patient and her family (63).

In this context, the treatments most favorite in the recent pediatric guidelines vary by efficacy and are not exempt from side effects. Specifically in mild-to-moderate pediatric asthma, we have several comparative studies for different add-on strategies such as increasing the doses of ICS (64), adding LABA, LAMA, or montelukast [reviewed in Vogelberg et al. (65)]. The comparisons among the aforementioned drugs and chromones or methylxanthines received less attention; however, a systematic review of asthma therapy for 5–18 year olds concluded that, although direct comparisons are lacking, there is no reason for considering theophylline inferior to LABA or LTRA (66). Although the paucity of further studies still makes it impossible to establish direct comparisons, indirect comparisons may be performed when planning an individual treatment. The performance characteristics of the different therapeutic strategies are multifaceted, but in some cases, an add-on therapy should include a methylxanthine. In our opinion, this could be considered at least in the following situations:

• risk of low compliance with inhalers (20, 67);
• risk of neurologic side-effects with LTRA (68);
• risk of serious asthma-related events attributable to LABAs (69, 70);
• risk of discontinuation due to perceived inefficacy with ICS/LABA (20);
• doubts on the efficacy of LAMA, due to paucity of pediatric data (71);
• need for concomitant use of drugs that interfere with cytochrome p450, as some antibiotics, anti-epileptics, and antiarrhythmics (35, 36).

Re. steroids themselves, alarm has been raised about the possibility of side effects in adults.

**FIGURE 5:**
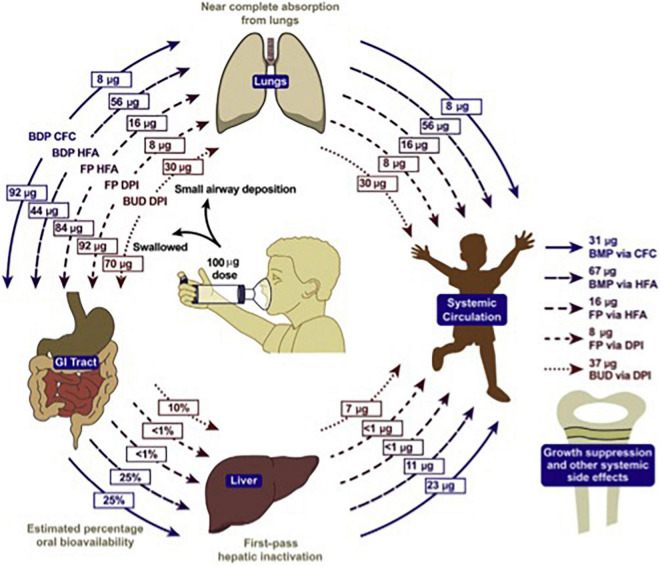
Systemic bioavailability of various ICS preparations (74).

Diabetes, obesity, osteopenia, osteoporosis, dyspeptic disorders, psychiatric disorders, and hypertension require a heavy toll when steroids are consumed orally (72). The cost is so significant that it can be compared to that of biological therapies (73, 74). In children, inhaled steroids may determine similar side effects when significantly absorbed, as it is the case for bechlometasone dipropionate (Figure 5) (75).

For this reason, limits have been established above which the continued use of inhaled corticosteroids in children is not considered prudent (Table 2) (2). Observing them, it is evident that some of the most widely used preparations for the prevention of asthma in children, especially in countries where the use of traditional aerosols is preferred, heavily exceeds the suggested limits (76).

**TABLE 2:** Limit dose for adeguate safety of the inhaled corticosteroids for age group (2).

Inhaled corticosteroid	Low total daily dose (mcg) (age group with adeguate safety and effectiveness data)		
**(A)**			

BDP (pMDI, standard particle, HFA)	100 (Ages 5 years and older)		
BDP (pMDI, extrafine particle, HFA)	50 (Ages 5 years and older)		
Budesonide nebulized	500 (Ages 1 years and older)		
Fluticasone propionate (pMDI, standard particle, HFA)	50 (Ages 4 years and older)		
Fluticasone furoate (DPI)	Not sufficiently studied in children 5 years and younger		

**(B)**			

**Children 6–11—see note above (for children 5 years and younger see box A)**			
Beclometasone dipropionate (pMDI, standard particle, HFA)	100–200	>200–400	>400
Beclometasone dipropionate (pMDI, extrafine particle, HFA)	50–100	>100–200	>200
Budesonide (DPI)	100–200	>200–400	>400
Budesonide (nebules)	250–500	>500–1,000	>1,000
Ciclesonide (pMDI, extrafine particle, HFA)	80	>80–160	>160
Fluticasone furoato (DPI)	50		n.a.
Fluticasone propionate (DPI or pMDI standard particle, HFA)	50–100	>100–200	>200

## Conclusion

When from a clinical examination it emerges that the proper addition therapy to a topical steroid can be a methylxanthine, the characteristics of efficacy and tolerability of doxofyllineundoubtedly place it as a better alternative than theophylline. The availability in sachet formulation makes the drug manageable and could encourage the improvement of therapeutic compliance, even more in the pediatric population. Many studies support its use in adult age; doxofylline lacks data in the pediatric field. Evaluation in real life of adherence to therapy, steroid sparing effect, and efficacy are needed in order to ensure the fair dignity to this drug. With this in mind, indirect comparisons with other add-on treatments may warrant the use of doxofylline in children.

## Author Contributions

VF and AF participated in the conception and drafting of the manuscript. AP and EB participated in the drafting of the manuscript. All authors contributed to the article and approved the submitted version.

## Conflict of Interest

VF received a fee for the drafting of the review. EB was employed by the ABC Farmaceutici S.p.a. The remaining authors declare that the research was conducted in the absence of any commercial or financial relationships that could be construed as a potential conflict of interest.

## Publisher’s Note

All claims expressed in this article are solely those of the authors and do not necessarily represent those of their affiliated organizations, or those of the publisher, the editors and the reviewers. Any product that may be evaluated in this article, or claim that may be made by its manufacturer, is not guaranteed or endorsed by the publisher.

## Abbreviations

CYP: Cytochrome P450
GINA: Global Initiative on Asthma
HDAC: Histone Deacetylase
ICS: Inhaled Corticosteroids
LABA: Long-Acting β -Agonists
LAMA: Long-Acting, Muscarinic Antagonists
LTRA: CysLTR1 Antagonists
PDE: Phosphodiesterase
SABA: Short-Acting β -Agonists.
